# The Cytokine Growth Differentiation Factor-15 and Skeletal Muscle Health: Portrait of an Emerging Widely Applicable Disease Biomarker

**DOI:** 10.3390/ijms232113180

**Published:** 2022-10-29

**Authors:** Boel De Paepe

**Affiliations:** Neuromuscular Reference Center, Ghent University Hospital, Corneel Heymanslaan 10, 9000 Ghent, Belgium; boel.depaepe@ugent.be

**Keywords:** growth differentiation factor-15, biomarker, muscle disorders

## Abstract

Growth differentiation factor 15 (GDF-15) is a stress-induced transforming growth factor-β superfamily cytokine with versatile functions in human health. Elevated GDF-15 blood levels associate with multiple pathological conditions, and are currently extensively explored for diagnosis, and as a means to monitor disease progression and evaluate therapeutic responses. This review analyzes GDF-15 in human conditions specifically focusing on its association with muscle manifestations of sarcopenia, mitochondrial myopathy, and autoimmune and viral myositis. The use of GDF-15 as a widely applicable health biomarker to monitor muscle disease is discussed, and its potential as a therapeutic target is explored.

## 1. Introduction

Skeletal muscle is the most abundant tissue in the human body and accounts for some 40% of our body mass. As maintaining voluntary movement is vital to our survival, muscle possesses impressive adaptive and regenerative capacities mediated by the expansion and differentiation of quiescent muscle progenitors termed satellite cells. In these processes of tissue recovery, the immune system plays a key role engaging a tightly orchestrated regenerative immune response. At the earliest stage of muscle injury, disintegration of muscle cell membranes releases cellular contents and chemotactic factors into the extracellular space, recruiting immune cells to the injury site. At this stage, infiltrating cytotoxic immune cells clear damaged muscle fibers and debris, and release cytokines, further activating and recruiting immune cells. In a second stage, a cascade of tissue-remodeling processes is initiated, mediated by a different subset of immune cells. Macrophages play a leading role in this conversion of the pro-inflammatory microenvironment at the site of injury to an anti-inflammatory microenvironment supportive of muscle recuperation [1]. Satellite cells follow a program of activation, differentiation, fusion, and maturation to form new muscle tissue. Successful regeneration restores damage to muscle fibers, but an imbalance between anabolic and catabolic pathways in favor of the latter may lead to loss of muscle mass and replacement with fibrotic and fatty tissue [2]. Skeletal muscle’s regenerative capacities decrease with age and associate with human (muscle) disorders.

Growth differentiation factor 15 (GDF-15), a divergent member of the transforming growth factor-β (TGF-β) superfamily, is involved in a variety of biological processes. The cytokine has pleiotropic functions in different tissues and organs, hence the many names it carries: nonsteroidal anti-inflammatory drug-activated gene-1 (NAG-1), macrophage inhibitory cytokine-1 (MIC-1), placental bone morphogenic protein (PLAB), placental transformation growth factor-β (PTGF-β), and prostate derived factor (PDF). Recent insight led to its reclassification as a member of the glial cell-derived neurotropic factor (GDNF) family activated through rearranged during transfection (RET) [3]. GDNF family receptor alpha like (GFRAL) is its only known high-affinity receptor. Studies of GFRAL knockout mice reveal a homeostatic role, with GDF-15/GFRAL interactions limiting the weight gain in animals on a high-fat-diet [4]. The wide variety of cell types that can be persuaded to secrete GDF-15 in response to a broad range of stressors contrasts strongly with the restricted distribution of GFRAL, of which expression is limited to the hindbrain [5]. Activities of GDF-15 unrelated to central regulation remain ill-understood and might originate from interactions with soluble forms of GFRAL that lack the transmembrane anchoring domain, or alternative effects exerted in absence of GFRAL for instance through receptors of the TGF-β/Smad signaling pathway. GDF-15 is transcribed as pro-GDF-15 which can reside in the nucleus and in the cytoplasm and can form dimers. As a consequence, multiple forms of GDF-15 can be present within cells alongside the cleaved mature dimer, including pro-GDF15 monomer and pro-GDF15 dimer [6]. The activity of GDF-15 is regulated by intracellular translocation and secretion mediated through its N-terminal signal peptide. Secreted GDF-15 is an easily accessible and stable biomarker currently under development for human medicine. GDF-15 levels can be determined in serum and plasma samples using enzyme-linked immunosorbent assay technologies, and several commercial kits have become available already. Circulating levels of GDF-15 are high at birth, but quickly decrease and remain low in healthy individuals. No standard values have been proposed for clinical purposes yet, but explorative studies report values ranging between 300 and 600 pg/mL in healthy controls. A temporary increase is noted in pregnant women (originating from placental expression), as is a steady increase with advancing age. Additionally, lifestyle and environmental factors may lead to subtle increases of blood GDF-15 levels [7,8,9].

## 2. GDF-15 and Skeletal Muscle

Circulating levels of GDF-15 dramatically increase in a wide range of diseases without there being a singular predominant common denominator. A tight connection between GDF-15 and multiple pathogenic processes has been established and include chronic inflammation, tissue apoptosis, mitochondrial dysfunction, and oxidative stress. Increased levels of GDF-15 have been observed in many human conditions in which muscle function is affected, as part of tissue stress responses. It remains uncertain whether GDF-15 induction is mostly of benefit or detrimental to muscle tissue recovery. For murine skeletal muscle, a direct catabolic effect on muscle cells has been observed in vivo [4,10] and in vitro [11]. GDF-15 appears to stimulate TGF-β-pathway-mediated fibrotic changes via downregulation of muscle-protective microRNAs. However, a positive effect of GDF-15 on myoblast proliferation has also been put forward, mediated by activation of regeneration-promoting programs in macrophages [12]. Under the control of peroxisome proliferator-activated receptor γ (PPARγ) and retinoid X receptor α (RXRα), GDF-15 is co-expressed with well-known muscle regeneration-associated growth factors, and is expressed specifically in a subpopulation of macrophages involved in muscle repair. Such contradictory activities of the same cytokine could be explained by beneficial effects mediated by cell-specific and timely expression that might activate very different mechanisms compared to the effects mediated by systemic upregulation. Indeed, chronically elevated circulating GDF-15 levels trigger adversary effects on growth and metabolism and mark a higher risk of all-cause mortality [13]. This review will give a general and concise roundup of the associations of GDF-15 with declining muscle health and evaluate its potential as a muscle disease biomarker and therapeutic target.

## 3. Age-Related Sarcopenia

From age 40 years onward, human skeletal muscle quantity and quality may decline progressively [14]. Such progressive and generalized loss of muscle mass and function is termed sarcopenia and represents a multifactorial process in which inadequate synthesis of muscle proteins plays a key role. Muscle protein synthesis is regulated through Akt mammalian target of rapamycin (mTOR) activation, under the control of growth factors including testosterone and insulin-like growth factor 1 (IGF-1) and is stimulated by exercise [15]. During the aging process, anabolic hormone levels decline, which leads to decreased muscle protein synthesis and subsequent loss of muscle mass. Sarcopenia compromises physical capacities of the growing population of people with an advanced age and is aggravated further by lifestyle factors including inactivity and poor nutrition [16]. Muscle impairment in the elderly results in progressive dependency and increases their risk of falls, fractures, and morbidities. Recognition of sarcopenia at an early stage allows taking measures to slow down progression and avoid excessive muscle loss. Interventions include resistance training, and nutraceuticals and drugs that stimulate muscle mass and strength building [17].

As a stress-induced cytokine, GDF-15 is considered a marker of biological age [18]. In aging, impaired mitochondrial function is a strongly associated mechanism, causing accumulation of reactive oxygen species and subsequent oxidative stress-induced tissue damage. While steady increases of circulating GDF-15 with advancing age may not be pathological, more pronounced elevation of GDF-15 exhibits an adversary effect on muscle mass, and associates with decreased muscle strength [19]. From mouse models, we know that increasing serum and muscle levels of GDF-15 in aging mice leads to reduced food intake, weight loss, and reduced skeletal muscle mass and function [20]. The muscle tissue itself actively contributes to the elevation of circulating GDF-15. Murine myotube cultures can be stimulated to produce GDF-15 by oxidative and endoplasmic reticulum stressors that mimic age-related stress [21]. While a firm and consistent association of GDF-15 with muscle catabolism comes forward in animal studies, it is less so in human studies. Some studies link sarcopenia, frailty, and reduced physical performance in older individuals with higher levels of circulating GDF-15 [20,22,23], other studies report gender-specificity [24] or fail to observe an association [25,26], or report that GDF-15 levels cannot predict sarcopenia [27]. Further studies are needed to confirm or refute if measuring circulating GDF-15 levels is helpful to recognize sarcopenia early in elderly individuals.

## 4. Disease-Related Sarcopenia

Muscle wasting associates with many human diseases and finds its origin in a multitude of mechanisms, which include oxidative stress, systemic inflammation, increased activity of the ubiquitin proteasome pathway, apoptosis, and/or an impaired regenerative potential of muscle [28], and is aggravated by disuse and malnutrition. In a multitude of human diseases, circulating GDF-15 levels are strongly increased, with levels negatively correlating with lean body and muscle mass [29] and positively correlating with patient fatigue scores [30]. The relationship between GDF-15 and pathologies that associate with sarcopenia will be discussed in this review.

### 4.1. Cardiovascular Disease

Heart disease accelerates the loss of muscle mass and severely compromises patients’ cardiorespiratory fitness and physical performance. Vice versa, sarcopenia may induce cardiovascular incidents [31]. Common contributing factors are physical inactivity, insulin resistance and malnutrition [32]. GDF-15 blood levels are extensively being explored as a cardiovascular disease marker indicative of metabolic health, and are reported to be increased in ischemia, heart failure, and hypertrophic and dilated cardiomyopathy [33,34], and in patients with type 2 diabetes at greater risk of heart failure [35]. GDF-15 is expressed in atherosclerotic lesion in arteries and seems actively involved in their formation and progression [36]. Circulating GDF-15 levels reflect renal dysfunction and muscle wasting in preoperative cardiovascular surgery patients [37].

For muscle health, preventive medication with statins, widely used to reduce cardiovascular risk, is a point of concern. These agents lower blood cholesterol levels by inhibiting 3-hydroxy-3-methylglutaryl coenzyme A reductase and, in addition, have beneficial immunomodulatory effects [38]. However, statins have also been linked to adverse effects of muscle damage and pain, and even rhabdomyolysis [39]. Although statin use is perceived as a probable risk for developing sarcopenia, the pros of cholesterol lowering drugs outbalance the possible risks. In addition, a study in heart failure patients observes, unexpectedly, that patients on statins develop sarcopenia less frequently than untreated patients [40]. Statins do not seem to influence GDF-15 levels, as is observed in diabetes type 2 patients on atorvastatin medication [41].

### 4.2. Chronic Inflammatory Diseases

In disorders characterized by chronic inflammation of lung and bowel, sarcopenia often co-exists and negatively impacts disease morbidities and mortality.

Chronic obstructive pulmonary disease (COPD) is one of the leading causes of morbidity and mortality worldwide with ever increasing importance [42], and the prevalence of sarcopenia in patients is high [43]. Muscle wasting is accompanied by weakness and fatigue and a slow-to-fast shift in fiber type composition [44]. Increased levels of GDF-15 are firmly linked with COPD. The cytokine is involved in the buildup of pulmonary inflammation, and levels predict disease outcomes in patients [45,46]. Circulating GDF-15 levels negatively associate with skeletal muscle mass and strength in patients [47]. The allocated diagnostic performance of GDF-15 as a serum marker for sarcopenia in COPD is of 70% sensitivity and 90% specificity, with an area under the curve (AUC) of 0.83 [48].

The chronic inflammatory bowel diseases ulcerative colitis and Crohn’s disease (CD) exhibit similar symptoms of digestive dysfunction and inflammation. Disease symptoms may include weight loss, with an average of 1 in 10 patients suffering from sarcopenia [49]. Muscle wasting develops via a multifactorial process involving chronic inflammation, malabsorption of nutrients by the damaged intestine, disuse, and (glucocorticoid) therapy [50]. Serum levels of GDF-15 are increased in CD and are significantly higher in patients with low skeletal muscle mass index [51].

### 4.3. Cancer

Frailty due to progressive loss of body weight, fat, and muscle, termed cachexia, is very common in cancer patients and responsible for approximately 1 in 4 cancer deaths. In some cancers, the risk of developing cachexia is very high, for example as high as 70% in pancreatic cancers, while low incidence is observed in other cancers, for example in melanoma [52]. Underlying mechanisms are energy imbalance caused by reduced food intake, an inflammation-driven shift from anabolic to catabolic metabolic processes and impaired muscle protein translation [53,54]. In addition, chemotherapy may aggravate the loss of muscle mass and strength, compromising treatment dosage and continuation [55]. The important role GDF-15 plays in cancer is illustrated further by the association of gene variants of the cytokine with cancer risk [56], prognosis [57], and mortality [58].

The proposition of GDF-15 as a useful biomarker for cancer is long-standing [59]. GDF-15 is actively involved in cancer progression and invasion [60] and impacts the tumor immune environment via mitogen-activated kinase activities [29]. The cytokine is actively secreted by tumors, produced by the cancer cells themselves or by tumor-associated macrophages [61]. GDF-15 potentially has both protective and tumor-promoting activities, inhibiting tumor growth in the early stages while inversely promoting tumor cell proliferation at later stages via metabolic and immunomodulatory mechanisms [62]. Evidence accumulates of a positive association between GDF-15 levels and cancer-induced cachexia [4,29]. Animal models appoint GDF-15 with a direct metabolic effect via loss of appetite and subsequent weight loss [63]. In analogy with its emetic effect during pregnancy [64], GDF-15 is observed to also contribute to chemotherapy-induced nausea and vomiting [65].

## 5. Mitochondrial Myopathy

Inherited defects that cause mitochondrial dysfunction lead to impaired oxidative phosphorylation and subsequent energy-deficits. Mitochondrial diseases are notoriously heterogeneous with onset at any age and affecting any part of the body [66]. The genetic origin of defects is complex, as cellular factors required for mitochondrial functioning lie encoded in both the nuclear and the mitochondrial DNA, the latter being present in multiple copies and possibly in heteroplasmic state. This complexity of inheritance seriously complicates identification of the causal gene defect and prediction of disease severity [67]. Tissues with high energy demands are first victims, hence many mitochondrial defects associate with prominent muscular problems.

Due to a firm association of the cytokine with mitochondrial dysfunction, GDF-15 blood levels are now a well-established diagnostic biomarker for mitochondrial diseases [68,69,70,71,72]. Its performance is, however, varied and dependents on the nature of the genetic defect and the disease presentation. GDF-15 blood levels appear to be especially useful to diagnose early onset mitochondrial myopathy, and to identify mtDNA deletions and translation defects. Affected muscle cells in muscle-manifesting mitochondrial disorders actively express and release GDF-15 [68,73], leading to increased circulating levels of the cytokine in patients. In a cohort of 48 patients diagnosed with mitochondrial encephalomyopathy, lactic acidosis, and stroke-like episodes (MELAS), Leigh syndrome or Kearns-Sayre syndrome (KSS), serum GDF-15 had an impressive diagnostic sensitivity and specificity nearing 100% and AUC of 0.997 [69]. In a more diverse group of 16 patients with molecularly confirmed mitochondrial disorders, serum GDF-15 had a sensitivity of 70% and specificity of 90% and an AUC of 0.82. Highest levels of GDF-15 were present in the serum of patients with thymidine kinase 2 (TK2) defects, MELAS, defects in *MT-TL1* encoding mitochondrial tRNA leucine, and patients with Pearson syndrome and KSS [70].

## 6. Autoimmune Myositis

Myositis of autoimmune origin is a heterogeneous group of rare disorders. Among its main subgroups recognized today are immune-mediated necrotizing myopathy (IMNM), dermatomyositis (DM), sporadic inclusion body myositis (IBM), polymyositis (PM), and myositis as part of the anti-synthetase syndrome [74]. For further subtyping of patients into categories relevant to prognosis and therapeutic outcome, an expanding list of myositis-specific and myositis-associated auto-antibodies is available [75]. IBM is the most common form of autoimmune myositis in patients above the age of 50 years [76]. IBM patients develop degenerative changes of skeletal muscle comparable to those associated with aging however at an accelerated pace, which include mitochondrial dysfunction and disturbed mitophagy [77]. In analogy with mitochondrial myopathies, elevated levels of GDF-15 are detected in IBM patients [78,79]. Mitochondrial dysfunction is, however, a more general aspect of the disease mechanisms underlying autoimmune myositis, and is also noted in other subgroups of myositis patients. Abnormal mitochondrial activities have been observed in the atrophic perifascicular muscle fibers typically present in DM skeletal muscle biopsies [80]. Fitting with a more general involvement of mitochondrial dysfunction, increased GDF-15 levels are also indicative of DM, PM, and IMNM [81], however with lower sensitivities (70% versus 100%) and AUC (0.70 versus 0.92) in IMNM compared to IBM, respectively [82]. Another obvious mechanism responsible for the increased levels of GDF-15 in autoimmune myositis is related to the associated chronic inflammation and active regeneration of muscle fibers [78]. Although more studies are still needed, GDF-15 appears to be an attractive biomarker for further development, as it appears to be able to distinguish autoimmune myositis patients from those with hereditary disorders with secondary muscle inflammation [82].

## 7. SARS-CoV-2-Associated Myositis

Viruses can cause inflammation of skeletal muscle, with influenza- and entero-viruses most commonly reported [83]. Due to the ongoing coronavirus disease 2 (SARS-CoV-2) pandemic (COVID-19), SARS-CoV-2-associated viral myositis has, however, received much more attention recently. Myalgia, alongside respiratory manifestations and fever, features as a prominent symptom, and proximal muscle weakness is increasingly being reported in individual patients. The muscle involvement varies in severity from an asymptomatic elevation of creatine kinase (CK), a standard biomarker for muscle tissue damage, to severe rhabdomyolysis. An increasing number of patients with myositis attributable to SARS-CoV-2 are being described [84]. Myositis is mediated through different underlying mechanisms, which include angiotensin-converting enzyme (ACE) receptor-mediated direct entry of the virus and triggering of innate and adaptive immune activation and T-cell clonal expansion, perpetuated by the continuous production of pro-inflammatory cytokines [85].

Mitochondrial stress is a key factor for the severity of COVID-19 disease and for triggering the devastating symptoms associated with the lethal cytokine storm [86]. Skeletal muscle-related symptoms are common in both acute and post-acute patients [87], with varied presentation in individual patients. A subset of patients develops autoimmune myositis. SARS-CoV-2-induced classic DM has been reported, exhibiting the typical skin rashes and proximal muscle weakness [88]. Rarely, patients develop severe lung involvement in the presence of anti-melanoma differentiation-associated gene 5 (MDA5) antibodies [89], strongly resembling anti-MDA5-positive autoimmune myositis [90]. Higher levels of GDF-15 are being reported in COVID-19 patients, most notably in those with poor outcome [91,92,93,94]. The association of GDF-15 expression with muscle manifestations is yet to be investigated.

## 8. Discussion

Determining circulating GDF-15 levels is a powerful predictor of all-cause mortality [13,95,96]. It is an easily accessible surrogate marker of ill-health useful to detect inadequately controlled disease. As GDF-15 blood levels are inversely correlated with muscle mass and strength, the cytokine also represents a relatively inexpensive and low invasive biomarker for diagnosing or monitoring muscle disease manifestations. Additionally, its direct involvement in pathologic disease mechanisms could allow its exploration as a measure of therapeutic responses, or GDF-15 could be developed as a therapeutic target as such.

### 8.1. Diagnostic and Prognostic Biomarker

The need continues to improve (early) characterization of age-associated comorbidities. No blood-based biomarkers are currently available with acceptable predictability for sarcopenia, and no single biomarker can accurately evaluate severity. Different circulating markers are proposed as good candidates, including markers for inflammation and oxidative stress [97], and GDF-15 may be added to the list. Muscle manifestations are frequent in many human disorders, and GDF-15 serum levels reflect clinical characteristics and disease severity as seen in mitochondrial myopathies [68,69,70,71,72], but also in rheumatoid arthritis patients [98]. In cardiovascular disease, GDF-15 is also put forward as a prognostic biomarker. High serum levels of GDF-15 predict risk of mortality in patients admitted with myocardial infarction [99]. In elderly men, GDF-15 levels aid to better predict cardiovascular outcome as well as cancer mortality and morbidity [95]. GDF-15 levels have been suggested for identifying patients undergoing cardiovascular surgery at high risk of developing muscle wasting [37]. In autoimmune myositis, GDF-15 levels may be used to sub-classify patients and to stratify individuals into different clinical phenotypes [81,82]. From these observations, one can conclude that measuring GDF-15 in the blood represents an asset for patient disease management planning.

### 8.2. The Multi-Marker Approach

Elevated levels of GDF-15 are associated with many human conditions and disorders which obscures its diagnostic purpose. A multi-marker scheme where GDF-15 features along with other biomarkers could be deployed to improve diagnostic power. Such a strategy has been proposed for mitochondrial myopathies, where its combination with the other well-studied biomarker and regulator of energy metabolism fibroblast growth factor-21 (FGF-21) is proposed. Serum FGF-21 is described to perform well at identifying muscle-manifesting mitochondrial disorders, with sensitivities and specificities of 90% [100]. In a more diverse cohort of patients, of which the majority did not present with myopathy, specificity of 90% was confirmed, but lower sensitivity of 20% and an AUC of 0.69 were found [101]. As is the case for GDF-15, elevated circulating levels of FGF-21 does not specifically point to a diagnosis of mitochondrial disorders but can also be observed in other conditions, most notably in liver disorders [102]. Studies disagree which of the two performs better than the other for identifying patients with mitochondrial disorders, in favor of GDF-15 [102] or FGF-21 [103], and both biomarkers preferably identify muscle-manifesting disease [71]. Five studies that compared the performance of GDF-15 and FGF-21 as diagnostic biomarkers for mitochondrial myopathy all showed higher sensitivity of the former [69,70,71,104,105], three also allocated higher specificity to GDF-15 [69,104,105]. A positive correlation between the two cytokines is reported [70], but a recent study describes no such correlation [103]. Combining GDF-15 with FGF-21 might seem a good idea also for other diseases with prominent mitochondrial involvement, but intriguing FGF-21 levels are reported unchanged in IBM patients [79]. Other combinations with GDF-15 may also be explored. In evidence, combining GDF-15 with gelsolin plasma levels performed even better to differentiate patients with mitochondrial disease than the combination with FGF-21 (AUC 0.94 vs. 0.91) [106].

No single reliable biomarker for sarcopenia has been identified among the multitude of different disease contributors. The multifactorial nature of the condition makes exploring combinations of disease biomarkers a particularly useful strategy for diagnosis and follow-up. The combination of serum markers, diagnostic imaging, and functional testing has been proposed as an ideal biomarker panel [107]. X-ray and magnetic resonance imaging are able to provide objective and detailed quantification of muscle and fat-free mass. In the blood, alterations to the arsenal of cytokines, chemokines, and growth factors actively released by muscle cells, can be further explored for diagnosis of sarcopenia [108] or other muscle conditions. GDF-15 can feature in that scheme, in combination with other circulating stress-related factors inversely associated with muscle mass and function, including inducible heat shock protein 70 [109,110] and specific non-coding RNAs [111]. The search for susceptibility genes is another powerful tool to weigh the importance of the many factors involved in muscle disease, and genome-wide association studies in older individuals can identify gene variants that associate with muscle weakness [112]. Multi-omics approaches that combine the layers of knowledge obtained through genomics, transcriptomics and metabolomics, are powerful tools to unravel complex muscle disease mechanisms, as has recently been elegantly reviewed [113].

### 8.3. Therapeutic Response Biomarker and Treatment Target

Although individual aspects of GDF-15 activities are clearly emerging, the full impact this complex cytokine has on muscle health remains enigmatic. When physiological low levels become elevated, this leads to local effects in the muscle tissue and to systemic effects on metabolism, appetite and muscle mass, but we have yet to fully understand the what, the how and the when. High blood levels of GDF-15 and *GDF15* variants have, for instance, been linked to pregnancy-related nausea and vomiting [114], but increased GDF-15 levels do not seem to exhibit adverse effects in the majority of pregnant women [115]. The proliferation of scientific insight will eventually lead to the identification of putative molecular therapeutic targets for selective interference of deleterious actions of GDF-15 in pathological contexts while sparing its associated favorable effects [34].

As a systemic appetite suppressor, GDF-15 induces loss of muscle mass, but its influence on muscle function expands way beyond. From in vitro studies of muscle cells in culture, from in vivo studies in animal models, and from patient studies, the complex involvement of GDF-15 in muscle tissue buildup and muscle strength comes forward. However, an important additional regulatory aspect of GDF-15 is attributable to its immunomodulatory activities, protecting against excessive and prolonged inflammation [116]. GDF-15 stimulates oxidative phosphorylation in macrophages, repolarizing them toward their anti-inflammatory M2 phenotype [117], and inhibits dendritic cell maturation [118], classifying the cytokine as anti-inflammatory. Interestingly, nonsteroidal anti-inflammatory drugs increase GDF-15 expression in cancer cells [119]. The negative correlation of GDF-15 with CK levels in autoimmune myositis points to a possible protective effect against muscle catabolism [82].

Therapies can be perceived to promote these beneficial effects of GDF-15. Exercise, for instance, is beneficial to metabolic health and shown to increase circulating levels of GDF-15 [7]. The skeletal muscle tissue is a possible source, stimulated to produce GDF-15 by muscle contraction [120]. Irrespective of its precise involvements in pathogeneses, GDF-15 levels can be explored as a means to monitor disease and treatment response. GDF-15 blood levels reflect the disease course in TK2 deficiency, and decline in response to treatment with deoxynucleosides [121]. When designing appropriate therapeutic interventions, context-dependent adverse and beneficial effects of GDF-15 need to be identified and weighed before it may be considered as a therapeutic target (Table 1). The appetite-reducing effects of the cytokine for instance may be beneficial to patients with obesity but detrimental to anorexic patients.

In cancer, pleiotropic effects of GDF-15 are striking. The cytokine exhibits both positive and negative effects on metastasis [122], but the balance seems to tip over to the negative side. Evidence in animal models leads to believe that it is a feasible target to be explored for treating cachexia in advanced cancer. A rat model of cardiac cachexia shows a protective effect of a neutralizing monoclonal anti-GDF-15 antibody [123], and an antagonistic monoclonal anti-GFRAL antibody prevents RET activation and subsequent weight loss in tumor-bearing mice [124]. Clinical trials are ongoing exploring the therapeutic benefit of GFRAL and GDF-15 neutralizing antibodies in humans. In the NCT04068896 phase 1/2 dose-finding study with a GFRAL antagonist monoclonal antibody in combination therapy, blocking of GFD-15 signaling is evaluated in subjects with advanced solid tumors and pancreatic cancer. In the GDFATHER trial (NCT04725474), the possible anti-cachexia effects of a monoclonal anti-GDF-15 antibody in monotherapy or in combination with a checkpoint inhibitor is being evaluated in patients with advanced cancers [125].

## 9. Conclusions

Scientific evidence identifies GDF-15 as a multi-purpose biomarker not limited to any single disease or confined group of diseases. Circulating levels of the cytokine are induced by stress of various origin, with particularly strong association with mitochondrial dysfunction and chronic inflammation. Measuring GDF-15 in the blood represents a means to identify and quantify the muscle manifestations that occur in patients suffering from a variation of human disorders. Although not specific for any individual muscle disorder, measuring GDF-15 levels in the blood is a convenient and informative asset to the clinic with potential intervention-changing capacities.

## Figures and Tables

**Table 1:** Summary of the proposed ambiguous effects displayed by growth differentiation factor-15 in human disorders.

Adverse Effects	Human Disorders	Beneficial Effects
Anorexia fibrosis promotion	heart disease	cardioprotection metabolic reprogramming
loss of muscle mass	diet-induced obesity	appetite reduction lipolysis promotion
loss of muscle mass	diabetes	pancreatic β-cell protection metabolic reprogramming
loss of muscle mass fibrosis promotion	lung disease	immunomodulation
loss of muscle mass	mitochondrial disease	mitogenesis promotion energy homeostasis regulation
loss of muscle mass	myositis	muscle regeneration immunomodulation
cachexia metastasis promotion tumor immune evasion	cancer	immunomodulation metastasis prevention
